# Continuing decrease in coronary heart disease mortality in Sweden

**DOI:** 10.1186/1471-2261-14-9

**Published:** 2014-01-21

**Authors:** Johanna Berg, Lena Björck, Georgios Lappas, Martin O’Flaherty, Simon Capewell, Annika Rosengren

**Affiliations:** 1 Department of Medicine, Sahlgrenska University Hospital, Östra, c/o Annika Rosengren, CK Plan 2, SE-416 85 Gothenburg, Sweden; 2 Department of Molecular and Clinical Medicine, The Sahlgrenska Academy, University of Gothenburg, Gothenburg, Sweden; 3 Department of Public Health & Policy, Institute of Psychology, Health & Society. Whelan Building, Quadrangle, University of Liverpool, Liverpool L69 3GB, UK

**Keywords:** Myocardial ischemia, Mortality, Risk factors, Coronary heart disease

## Abstract

**Background:**

Deaths from coronary heart disease (CHD) have been decreasing in most Western countries over the last few decades. In contrast, a flattening of the decrease in mortality has been recently reported among younger age groups in some countries. We aimed to determine whether the decrease in CHD mortality is flattening among Swedish young adults.

**Methods:**

We examined trends in CHD mortality in Sweden between 1987 and 2009 among persons aged 35 to 84 years using CHD mortality data from the Swedish National Register on Cause of Death. Annual percent changes in rates were examined using Joinpoint software.

**Results:**

Overall, CHD mortality rates decreased by 67.4% in men and 65.1% in women. Among men aged 35–54 years, there was a modest early attenuation from a marked initial decrease. In the oldest women aged 75–84 years, an attenuation in the mortality decrease was observed from 1989 to 1992, followed by a decrease, as in all other age groups.

**Conclusions:**

In Sweden, coronary heart disease deaths are still falling. We were unable to confirm a flattening of the decline in young people. Death rates continue to decline in men and women across all age groups, albeit at a slower pace in younger men since 1991. Continued careful monitoring of CHD mortality trends in Sweden is required, particularly among young adults.

## Background

Cardiovascular disease is the leading cause of death in women in Europe and the main cause of death in European men, except in Spain, France, Israel, San Marino, Slovenia and the Netherlands [1]. In Europe, a north-east to south-west gradient has been reported where mortality from coronary heart disease (CHD) is lower in the south-west regions of Europe [2].

In the Western world, a decrease in CHD mortality has been observed in the last 20 to 30 years, as a result of population trends in risk factors, as well as improved treatment and prevention [3-5]. However, in the last 10 years, research has shown a flattening in the decrease in mortality among younger adults [6-10], or no decline at all among people aged less than 55 years [11]. In contrast, a study from the Netherlands showed an initial flattening of the mortality decline among young people followed by a decrease [12]. In Sweden, overall mortality rates from CHD have been declining since the 1980s [4]. Given that a flattening in the decline in CHD mortality in some countries has been demonstrated particularly among young people, the present study aimed to investigate whether this is occurring also in the Swedish population. Recent trends have not previously been reported for separate age groups in Sweden.

## Methods

We conducted a population-based observational study based on CHD mortality data from the Swedish National Register on Cause of Death, including the total population aged 35–84 years. Information on population count was acquired from the national board of Statistics Sweden. We limited our analysis to people aged 35–84 years and divided them into four groups: 35–54, 55–64, 65–74 and 75–84 years. To assign cause of death, we used the International Classification of Diseases (ICD)-9 codes 410–414 for 1987 to 1996 and ICD-10 codes I20-I25 for 1997 to 2009. We adjusted the death rates for age with the direct method [13] using the Swedish population of the year 2010.

To analyse the mortality rates over time, we used joinpoint regression for estimation of the annual percentage change and to identify the specific years when significant changes in the trends occurred (Joinpoint Regression Program, version 3.3.1. April 2008; Statistical Research and Applications Branch, National Cancer Institute). Joinpoint regression analysis was originally applied in cancer mortality analysis although recently also in CHD mortality trend analysis [8-10]. We fitted the data in a log-linear model and set the number of possible joinpoints between 0 and 3. The program analysed if there was a change in the trend curve, a suggested joinpoint where the annual percent change became inconstant. The true number of joinpoints was verified with a permutation test to obtain a significance level. For each estimate of mean annual percentage change, 95% confidence intervals (CIs) were calculated. Variance was analysed with the Poisson model using count. For confidentiality, all personal identity numbers were replaced by codes. The protocol was approved by the Ethics board of the University of Gothenburg.

## Results and discussion

### CHD mortality trends in the total population

**Figure 1:**
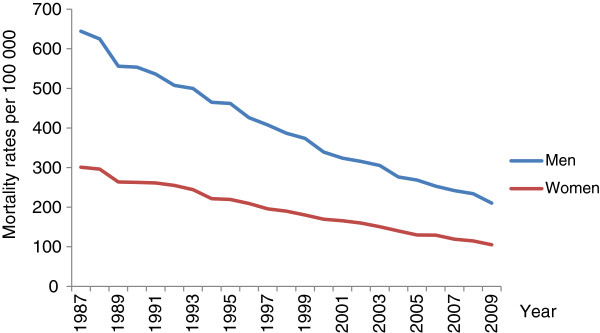
Trends in age-adjusted mortality rates from coronary heart disease mortality for adults aged 35–84 years in Sweden from 1987 to 2009.

In Sweden, the overall age-adjusted mortality rates from CHD decreased by 65.1% in women from 1987 to 2009 (Figure 1, Table 1). When analysed with joinpoint the average annual rate of decline over the 23-year period was 3.4% from 1987 to 1993 and 4.8% from 1993 to 2009. In men, the overall decrease in CHD mortality was 67.4% from 1987 to 2009 (Figure 1, Table 2). The average annual rate of decline was 4.2% from 1987 to 1995 and 5.1% from 1995 to 2009. Accordingly, the rate of decline of the overall CHD mortality rates has been more rapid in both sexes during the latter part of the period.

**Table 1:** Age-standardised trends in coronary heart disease mortality among Swedish women from 1987 to 2009: joinpoint analysis

**Age group (years) and periods**	**No of deaths (max-min)**^**1**^	**Rates per 10**^**5**^**(max-min)**^**2**^	**Annual percentage change(95% CI)**
35–54			
1987–2009	104–63	11.6 –5.2	-3.1* (-3.7 to -2.4)
55–64:			
1987–2009	482–216	107–39.1	-4.7* (-5.1 to -4.2)
65–74:			
1987–1996	1978–1346	417–289	-4.3* (-5.0 to -3.5)
1996–2009	1346–588	294–128	-5.7* (-6.3 to -5.2)
75–84:			
1987–1989	4928–4480	1617–1418	-6.5* (-10.9 to -1.8)
1989–1992	4518–4421	1418–1360	-1.5 (-6.4 to 3.7)
1992–2006	4421–2428	1360–729	-4.5* (-4.8 to -4.2)
2006–2009	2428–1855	729–578	-6.9* (-10.5 to -3.2)
35–84:			
1987–1993	7496–6376	301.1–244.2	-3.4* (-4.4 to -2.4)
1993–2009	6376–2758	244.2–105.1	-4.8* (-5.1 to -4.5)

*Annual Percentage Change (APC) is significantly different from zero at alpha = 0.05.

^1^The total number of deaths in the age group for each particular year.

^2^Comparison of the mortality rates in each age group and time interval.

**Table 2:** Age-standardised trends in coronary heart disease mortality among Swedish men from 1987 to 2009: joinpoint analysis

**Age group (years) and periods**	**No of deaths(max-min)**^**1**^	**Rates per 10**^**5**^**(max-min)**^**2**^	**Annual percentage change (95% CI)**
35–54:			
1987–1991	689–545	68.7–47.4	-8.1* (-11.5 to -4.6)
1991–2009	578–282	47.4–22.5	-3.9* (-4.4 to -3.4)
55–64:			
1987–2009	1992–796	465–132	-5.6* (-5.9 to -5.4)
65–74:			
1987–1997	5099–2923	1260–768	-4.6* (-5.1 to -4.1)
1997–2009	2923–1545	768–350	-6.1* (-6.6 to -5.6)
75–84:			
1987–1995	6284–5225	2927–2298	-3.1* (-3.9 to -2.4)
1995–2009	5320–2714	2299–1119	-4.6* (-5.0 to -4.2)
35–84:			
1987–1995	13935–10501	644.2–461.9	-4.2* (-4.7 to -3.6)
1995–2009	10501–5339	461.9–210.2	-5.1* (-5.4 to -4.8)

*Annual Percentage Change (APC) is significantly different from zero at alpha = 0.05.

^1^The total number of deaths in the age group for each particular year.

^2^Comparison of the mortality rates in each age group and time interval.

### Age-specific CHD mortality trends

**Figure 2:**
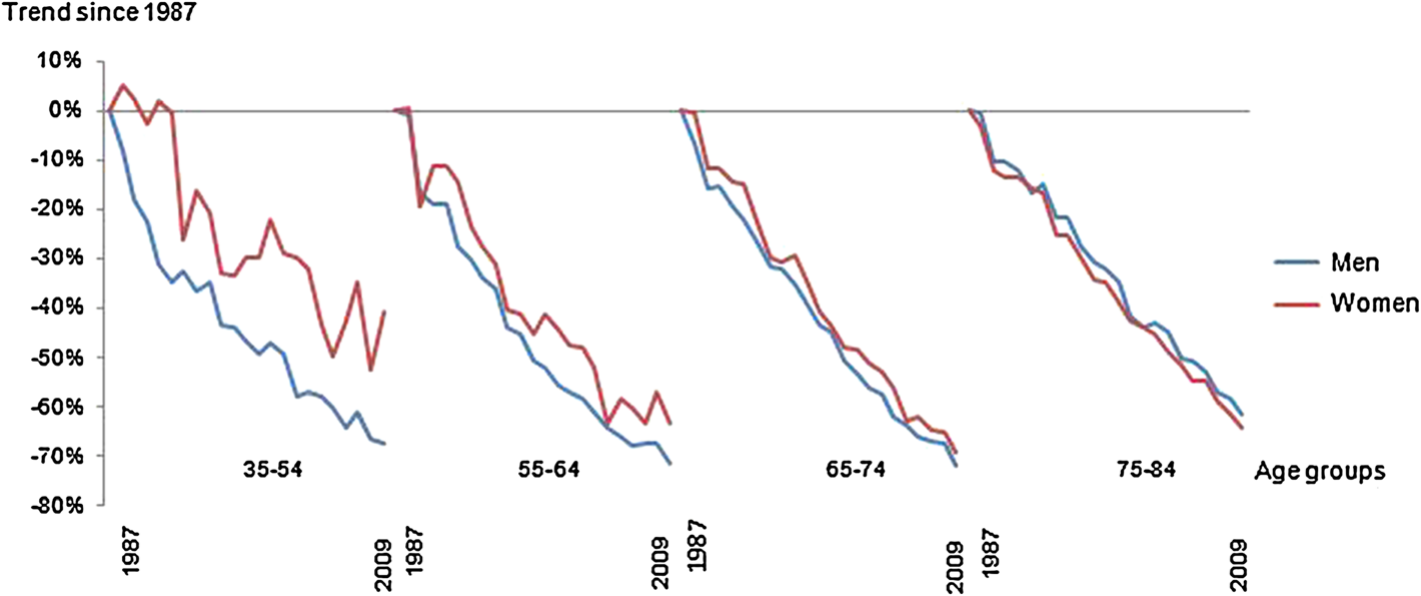
Trends in age-specific mortality rates from coronary heart disease mortality for adults aged 35–84 years in Sweden from 1987 to 2009.

When considering age-specific trends in mortality rates from 1987 among women aged 35–54 years, an increase was observed during the first few years, followed by a decrease (Figure 2, Table 1), and the rate varied from year to year. Apparent increases in mortality rates for young women for some years were observed, followed by decreases. The average annual change was 3.1% (95% CI: 3.7 to 2.4) from 1987 until 2009, with no significant joinpoint. In women aged 55–64 years, non-significant apparent attenuations were observed between 1989 to 1991, 1997 to 1999 and 2003 to 2005. Overall, the average annual change was 4.7% (95% CI: 5.1 to 4.2), with no significant change in the trend. The mortality continued to decline in 65–74 year old women, accelerating slightly after 1996. There was a brief flattening in the rate of decrease from 1989 to 1992 for the oldest women, after which mortality decreased.

Among the youngest men (35–54 years), there was a modest attenuation in CHD mortality rate between 1991 and 2009 from a very marked initial decrease (Figure 2, Table 2). In men aged 55–64 years, no flattening in mortality rate was observed. Among men aged 65–74 years and 75–84 years, both groups displayed joinpoints, with an accelerated decrease after the mid-90s.

In this study of the total Swedish population aged 35–84 years over a period of 23 years, we observed a continuing decrease in CHD mortality among men and women in all age groups over the last 2 decades, which is satisfactory. This is in contrast to some other countries, where a flattening in the decrease in CHD mortality has been observed among younger people [6-9,12]. Nevertheless, in the present study, a slower rate of decrease in CHD mortality was observed in men aged 35–54 years after 1991. In women aged 35–54 years, the age-adjusted mortality fluctuated, largely due to a limited number of events, with random variation, but there was no statistically significant change in the rate of annual change.

An estimation model has been used to estimate the impact of treatment and risk factors on CHD mortality [3-5,14]. Similar to findings from the UK, Ireland, the US, and other Western high-income countries, up to two thirds of the decrease in CHD mortality in Sweden from 1986 to 2002 can be explained by improved risk factors in the population (lower serum cholesterol, lower rates of smoking and lower blood pressure). The decrease in risk factors has resulted in a lower CHD incidence [15]. Improvement in medical interventions, including better treatment of acute myocardial infarction and secondary prevention can explain one third of the decrease in CHD mortality [4]. Because many fatalities occur outside hospital [16] in people with no known prior disease, the scope for improvement by improved care in the acute stage of the disease is limited.

The paradox of the decrease in CHD mortality observed in parallel with the ongoing obesity epidemic has been discussed [17,18]. Although obesity is linked to cardiovascular risk it seems to be associated with better survival. The paradox could partly be explained by a lower amount of risk factors even among the obese population [4,19]. It has further been suggested that the survival effects of obesity might be postponed through a gradual process of cardiac remodeling [20] or that obese patients present with CHD at a younger age and receive more effective treatment [21]. In Sweden, one-third of women and half of the men are overweight or obese, with obesity rates increasing in the last two decades [19,22], more among men than among women. Regardless, CHD mortality rates in Sweden are below those of many other countries [23]. This increasing obesity will probably lead to a raised incidence of diabetes and hypertension, increasing the risk factor burden. However, there may be a recent plateauing in the rate of obesity [24], and additionally, the incidence of type-2-diabetes mellitus and hypertension in the population has not appreciably increased [25-27]. Even so, there is a time lag between the incidence of obesity and CHD mortality and development of metabolic effects of obesity leading to atherosclerosis. Ultimately, if obesity trends in Sweden are not reversed, it appears unlikely that this reassuringly downward trend for CHD mortality for all ages in Sweden will continue.

The decline in CHD mortality in Sweden and other Western high income countries does not apply to all segments of the population. Data from 6 countries of Western Europe from the mid-80s to the mid-90s, including Sweden, show faster proportional mortality declines in higher socioeconomic groups, partly due to more rapid proportional mortality declines for cardiovascular diseases [28]. In the current study, we did not have data on social position, but other studies have shown a widening gap, at least with respect to population risk factors in Sweden [29]. Therefore, reducing these socioeconomic inequalities and providing access to prevention and modern treatment for all groups are crucial to secure a continuing decline in the mortality of CHD. Finally, continuing success in the battle against CHD is by no means ensured. Fad diets, with low carbohydrate – high fat content, have become increasingly popular in Sweden [30], and rising cholesterol levels after 2007 have recently been reported [31]. This finding warrants continued vigilance with respect to CHD mortality trends.

There are some limitations to the present study, including the reliance on data collected for administrative purposes, with a potential risk of misclassification. Recent findings from our group have highlighted the fact that 3 out of 4 CHD deaths occur out of hospital, and that this proportion is even greater among younger people [16]. Still, autopsy rates for out-of-hospital deaths are relatively high, particularly among younger subjects, where at least 80% of fatal cases are subjected to an autopsy, and accordingly, it is unlikely that misclassification could explain a lack of attenuation in CHD death rates. Additionally, the youngest age group, particularly women, comprises few deaths annually, which results in some fluctuations in the mortality trends that can be overstated. However, when these trends were analysed with joinpoint regression, they were not significant. The strengths of this study are that it covered the whole Swedish population for an extended period of time.

## Conclusions

In conclusion, over the last two decades, CHD mortality has been decreasing by approximately two-thirds in all age groups in Sweden. We were unable to confirm a flattening of the decline in young people. Prior research has shown that this is explained by improvements in risk factors in the population, subsequent lower incidence and by better medical treatment over a range of modalities. Unlike several other countries, Sweden, as yet, has not observed a clear flattening in these favourable trends. Despite this, it is apparent that decreasing trends are not guaranteed and recent reports on increasing cholesterol levels from 2008 and onwards are deeply concerning. Continued careful monitoring of CHD mortality trends in Sweden is required, particularly among young adults.

## Competing interests

The authors declare that they have no competing interests.

## Authors’ contributions

JB designed the study, performed the statistical analysis and prepared the manuscript. LB advised on the study design and interpretation of data. GL was responsible for data collection and statistical advice. MOF and SC provided statistical advice. AR advised on the study design and interpretation of data. All authors contributed to the study design, read, critically revised, and approved the final manuscript.

## Authors’ information

Johanna Berg, MD, PhD.

Lena Björck, PhD, Postdoctoral Research Assistant.

Georgios Lappas, MSc, Statistician.

Martin O’Flaherty, MD, PhD, Senior Research Officer.

Simon Capewell, MD, PhD, Professor of Clinical Epidemiology.

Annika Rosengren, MD, PhD, Professor of Medicine.

## Pre-publication history

The pre-publication history for this paper can be accessed here:

http://www.biomedcentral.com/1471-2261/14/9/prepub
